# The role of postmastectomy radiotherapy in clinically node-positive, stage II-III breast cancer patients with pathological negative nodes after neoadjuvant chemotherapy: an analysis from the NCDB

**DOI:** 10.18632/oncotarget.6664

**Published:** 2015-12-18

**Authors:** Jieqiong Liu, Kai Mao, Shuai Jiang, Wen Jiang, Kai Chen, Betty Y.S. Kim, Qiang Liu, Lisa K. Jacobs

**Affiliations:** ^1^ Guangdong Provincial Key Laboratory of Malignant Tumor Epigenetics and Gene Regulation, Breast Tumor Center, Sun Yat-sen Memorial Hospital, Sun Yat-sen University, Guangzhou, China; ^2^ Department of Surgery, Johns Hopkins University School of Medicine, Baltimore, MD, USA; ^3^ Guangdong Provincial Key Laboratory of Malignant Tumor Epigenetics and Gene Regulation, Department of General Surgery, Sun Yat-sen Memorial Hospital, Sun Yat-sen University, Guangzhou, China; ^4^ Department of Medicine, Johns Hopkins University School of Medicine, Baltimore, MD, USA; ^5^ Department of Epidemiology, Johns Hopkins Bloomberg School of Public Health, Baltimore, MD, USA; ^6^ Department of Radiation Oncology, MD Anderson Cancer Center, Houston, TX, USA; ^7^ Department of Neurological Surgery, Mayo Clinic Florida, Jacksonville, FL, USA

**Keywords:** breast cancer, postmastectomy radiotherapy, complete pathological nodal response, neoadjuvant chemotherapy, survival benefit

## Abstract

**Purpose:**

The role of postmastectomy radiotherapy (PMRT) in clinically node-positive, stage II-III breast cancer patients with pathological negative nodes (ypN0) after neoadjuvant chemotherapy (NAC) remains controversial.

**Methods:**

A total of 1560 clinically node-positive, stage II-III breast cancer patients treated with NAC and mastectomy who achieved ypN0 between 1998 and 2009 in the National Cancer Database were analyzed. The effects of PMRT on overall survival (OS) for the entire cohort and multiple subgroups were evaluated. Imputation and propensity score matching were used as sensitivity analyses to minimize biases.

**Results:**

Of the entire 1560 eligible patients, 903 (57.9%) received PMRT and 657 (42.1%) didn’t. At a median follow-up of 56.0 months, no statistical difference was observed for OS between two groups by univariate and multivariate analyses (*P* = 0.120; HR 1.571, 95% CI 0.839-2.943). On subgroup analyses, PMRT significantly improved OS in patients with clinical stage IIIB/IIIC disease, T3/T4 tumor, or residual invasive breast cancer after NAC (*P* < 0.05). This improvement in OS remained significant after sensitivity analyses for the propensity score-matched patients.

**Conclusions:**

This study demonstrated that PMRT showed a heterogeneous effect in clinically node-positive, stage II-III breast cancer patients with ypN0 following NAC. PMRT improved OS for patients with clinical stage IIIB/IIIC disease, T3/T4 tumor, or residual invasive breast tumor after NAC. In the absence of definitive conclusions from prospective studies, including the ongoing NSABP B-51 trial, our findings may help identify specific groups of women with clinically node-positive, stage II-III breast cancers who could benefit from PMRT after NAC.

## INTRODUCTION

The optimal patient selection criterion for postmastectomy radiotherapy (PMRT) in the management of breast cancer is a subject of ongoing debate. Previous randomized trials have established a clear guideline regarding the use PMRT in the setting of adjuvant chemotherapy [1–3]. However, whether PMRT can provide similar benefits in patients who had excellent pathological response after treatment with preoperative chemotherapy (NAC) is less clear. No results from prospective trials have been reported to evaluate PMRT's effect in the neoadjuvant setting. The available retrospective data suggest that the initial extent of disease clinically at presentation, the response of axillary lymph nodes to NAC, and the pathologic extent of residual disease are important factors to consider regarding the use of PMRT after NAC [4–6]. Thus, the National Cancer Institute (NCI) statement recommends that PMRT to the chest-wall and regional nodal basins should be considered for patients with clinical stage III disease or have histologically positive nodes after NAC [7]. Despite this, it remains unclear as to whether PMRT can provide improved patient outcomes for women with clinically node-positive, stage II to III breast cancer, but had a complete pathological nodal response (ypN0) after NAC. Previous studies aimed to address this question based on small retrospective cohorts have produced inconsistent results [4, 8–11]. A French group and another Korean study both showed that PMRT was not correlated with improved outcomes in clinical stage II-III patients with ypN0 after NAC [8, 10]. In contrast, research conducted at MD Anderson Cancer Center found that PMRT significantly improved local-regional recurrence (LRR) and cancer-specific survival (CSS) in clinical stage III breast cancer women even when they achieved a pathological complete response (pCR) following NAC [4, 11].

Therefore, there is a lack in consensus among practitioners regarding to the treatment recommendations of PMRT for clinically node-positive, stage II-III breast cancer patients with ypN0 after NAC. This was further demonstrated by a 2013 survey of 372 radiation oncologists which showed a split decision regarding treatment recommendations for clinical stage T2N1 patients who achieve ypN0 after NAC, with 49.9% of those surveyed recommending PMRT [12]. Given the conflicting results of small retrospective studies and lack of findings from randomized controlled trials, we analyzed a large national cohort of breast cancer patients, the National Cancer Database (NCDB), to identify the effectiveness of PMRT in terms of overall survival for clinically node-positive, stage II-III breast cancer patients with ypN0 after NAC.

## MATERIALS AND METHODS

### Patient population

We used data from the NCDB, which is a national hospital-based cancer registry jointly sponsored by the American College of Surgeons and the American Cancer Society, and collects data on about 70 % of newly diagnosed breast cancer cases in the United States. Data are coded and reported according to nationally established protocols coordinated under the auspices of the North American Association of Central Cancer Registries.

Data within the NCDB were rendered anonymous, so the study was exempt from review by the Johns Hopkins Medicine Institutional Review Board, and no consent was needed in this study.

A total of 2,807,541 breast cancer cases (International Classification of Diseases for Oncology, 3rd edition [ICD-O-3] histology codes 8000-8576, 8980-8981, and 9020/3 [13]), diagnosed between 1998 and 2012, were identified. The inclusion criteria were women 18 years or older, clinically node-positive and stage II-III (AJCC) breast cancer, treated with NAC and mastectomy with pathologically confirmed complete nodal response (ypN0). To ensure adequate follow-up time, we included cases diagnosed from 1998 through 2009. The timing of chemotherapy is coded in the NCDB as a temporal sequence with relation to definitive surgical therapy, allowing the accurate identification of NAC. Patients with positive or unknown surgical margin, pathological tumor size > 5 cm after NAC, distant metastatic disease, or prior malignancy were excluded. Additional exclusion criteria included unknown clinical or pathological tumor/node stage, preoperative or intraoperative radiotherapy, or radiotherapy not for chest wall and draining lymphatics. This resulted in a cohort of 1580 patients of which 907 received PMRT and 673 patients did not. The primary endpoint for this study was overall survival (OS), which is defined as the time from diagnosis to the date of death from any cause. Some patients did not receive radiotherapy after surgery because of rapid death (due to disease progression or post-operative complications) or loss of follow-up. To minimize this potential bias between groups, we excluded patients who died or lost to follow-up within 3 months after mastectomy: 4 (0.4%) in the irradiated group and 16 (2.4%) in the non-irradiated group. This left 1560 patients for final analysis.

### Statistical analysis

#### Primary analysis

Characteristics of the entire study population were presented according to PMRT treatment. The demographic and clinicopathological characteristics were compared between the two groups using the χ^2^ test. OS curves were constructed using the Kaplan-Meier method and compared between the two groups using the log-rank test. Multivariate Cox proportional hazard model was applied to assess the independent prognostic effect of PMRT or other factors. Likelihood ratio test was used to select the best multivariate Cox model. We also performed subgroup analyses to identify the role of PMRT on OS in various subgroups of patients.

#### Sensitivity analysis using imputation and propensity score

Propensity score-based sensitivity analysis was done to minimize selection bias or a lack of covariate balance. In the NCDB database, some key variables (eg. histologic grade) contain missing data, which may result in biases. To compensate for this, multiple imputation methods by chained equations [14–16] to account for the missing values of several variables was performed before the propensity score matching. NCDB has neither ER/PR records before 2004, nor Chalson/Deyo score before 2003, we can not assume unknown ER/PR status and Chalson/Deyo score are missing at random. Thus, we conducted the imputation to accommodate missing data for insurance status, histologic grade, number of examined lymph nodes, chemotherapy type, and use of hormone therapy, but not for ER/PR status or Chalson/Deyo score. A probabilistic rule based on regression models for each covariable with the other covariables serving as predictors was used to impute possible values for individual missing values. A full dataset was created after imputing for 10 times using “complete” function in MICE package [16, 17].

For the entire study cohort and individual subgroups, we performed logistic regression to select demographic and clinicopathological variables associated with the implementation of PMRT. All variables with a univariate *P* value ≤0.20 were eligible for inclusion in the logistic regression model. The final multivariate logistic model was used to calculate the propensity score for each individual, which is the probability of the patient being treated with PMRT. Patients who received PMRT were matched to patients who did not receive PMRT by propensity score ± 0.1 in a 1:1 ratio. The quality of the matching was checked by calculating the standardized difference for each covariate, assuming that the balance was achieved if the standardized difference was less than 0.1 [18]. Univariate and/or multivariate survival analyses were performed in the propensity score-matched populations using the same methods as those in the primary analysis.

Statistical analyses were conducted using STATA 12.0 software (StataCrop, College Station, TX) or R software (R Core Team 2014 [19]). All statistical tests were two-sided, and statistical significance was defined as *P* < 0.05.

## RESULTS

### Patient and treatment characteristics

Of the 1560 clinically node-positive, stage II-III breast cancer patients who had complete pathological nodal response after NAC and mastectomy, 903 (57.9%) received PMRT and 657 (42.1%) did not. All the patients had negative surgical margins. Table 1 presents the comparisons of demographic, clinicopathological, and treatment characteristics between these two cohorts of patients. When compared with patients who did not receive PMRT, irradiated patients had less comorbidities, more advanced clinical tumor stage, nodal stage, or AJCC stage, more regional lymph nodes examined, and less unknown ER/PR status, and received more multi-agent chemotherapy or hormone therapy (*P* < 0.01 for all comparisons). No difference was found between the two groups with respect to age, race, insurance status, pathological tumor stage (after NAC), or histologic grade. For the patients treated with PMRT, radiation targets included chest wall and draining lymphatics, with or without a chest wall boost. The median dose of radiation was 50.4 Gy.

**Table 1:** Characteristics of the whole study population (*n* = 1560)

Characteristics	No PMRT (*n* = 657)			PMRT (*n* = 903)		*P*
	No.	%	No.		%	
Age, years						NS
Median (range)	50 (20-86)			50 (22-88)		
≤40	143	21.8		203	22.5	
41-60	386	58.7		545	60.3	
>60	128	19.5		155	17.2	
Race						NS
White	494	75.2		693	76.7	
Black	121	18.4		162	17.9	
Asian or other	42	6.4		48	5.3	
Insurance status						NS
Not insured	31	4.7		49	5.4	
Private insurance	426	64.8		620	68.7	
Public insurance	186	28.3		227	25.1	
Unknown	14	2.1		7	0.8	
Chalson/Deyo score						<0.001
0	552	84.0		797	88.3	
1	44	6.7		83	9.2	
2	10	1.5		7	0.8	
Unknown	51	7.8		16	1.8	
Year of diagnosis						<0.001
1998-2003	90	13.7		56	6.2	
2004-2009	567	86.3		847	93.8	
Histological type						
Ductal	540	82.2		718	79.6	NS
Lobular	50	7.6		61	6.7	
Other	67	10.2		124	13.7	
No. of nodes examined						0.009
Median (range)	11 (1-46)			12 (1-46)		
1-10	317	48.2		379	42.0	
>10	320	48.7		507	56.1	
Unknown	20	3.0		17	1.9	
Clinical T-stage						<0.001
T1	79	12.0		55	6.1	
T2	276	42.0		254	28.1	
T3	170	25.9		279	30.9	
T4	132	20.1		315	34.9	
Clinical N-stage						<0.001
N1	530	80.7		651	72.1	
N2	90	13.7		161	17.8	
N3	37	5.6		91	10.1	
Clinical AJCC stage						<0.001
II	325	49.5		231	25.6	
III	332	50.5		672	74.4	
Pathologic T-stage (after NAC)						NS
T0/Tis	277	42.2		399	44.2	
T1	221	33.6		315	34.9	
T2	159	24.2		189	20.9	
Histologic grade						NS
Well or moderately	161	24.5		199	22.0	
Poorly or undifferentiated	413	62.9		613	67.9	
Unknown	83	12.6		91	10.1	
ER^*^						<0.001
Negative	330	50.2		503	55.7	
Positive	208	31.7		331	36.7	
Unknown	119	18.1		69	7.6	
PR^*^						<0.001
Negative	379	57.7		563	62.3	
Positive	159	24.2		270	29.9	
Unknown	119	18.1		70	7.8	
Chemotherapy type						<0.001
Single-agent	13	2.0		4	0.4	
Multi-agent	588	89.5		881	97.6	
Unknown if single or multi-	56	8.5		18	2.0	
Hormone therapy						<0.001
No	449	68.3		539	59.7	
Yes	181	27.5		335	37.1	
Unknown	27	4.1		29	3.2	

Abbreviations: PMRT, postmastectomy radiotherapy; NS, not significant (*P* >0.05);

AJCC, American Joint Committee on Cancer (fifth or sixth edition); NAC, neoadjuvant chemotherapy; ER, estrogen receptor; PR, progesterone receptor

* ER or PR groups include those with borderline results.

### Survival analyses for the whole population

**Figure 1:**
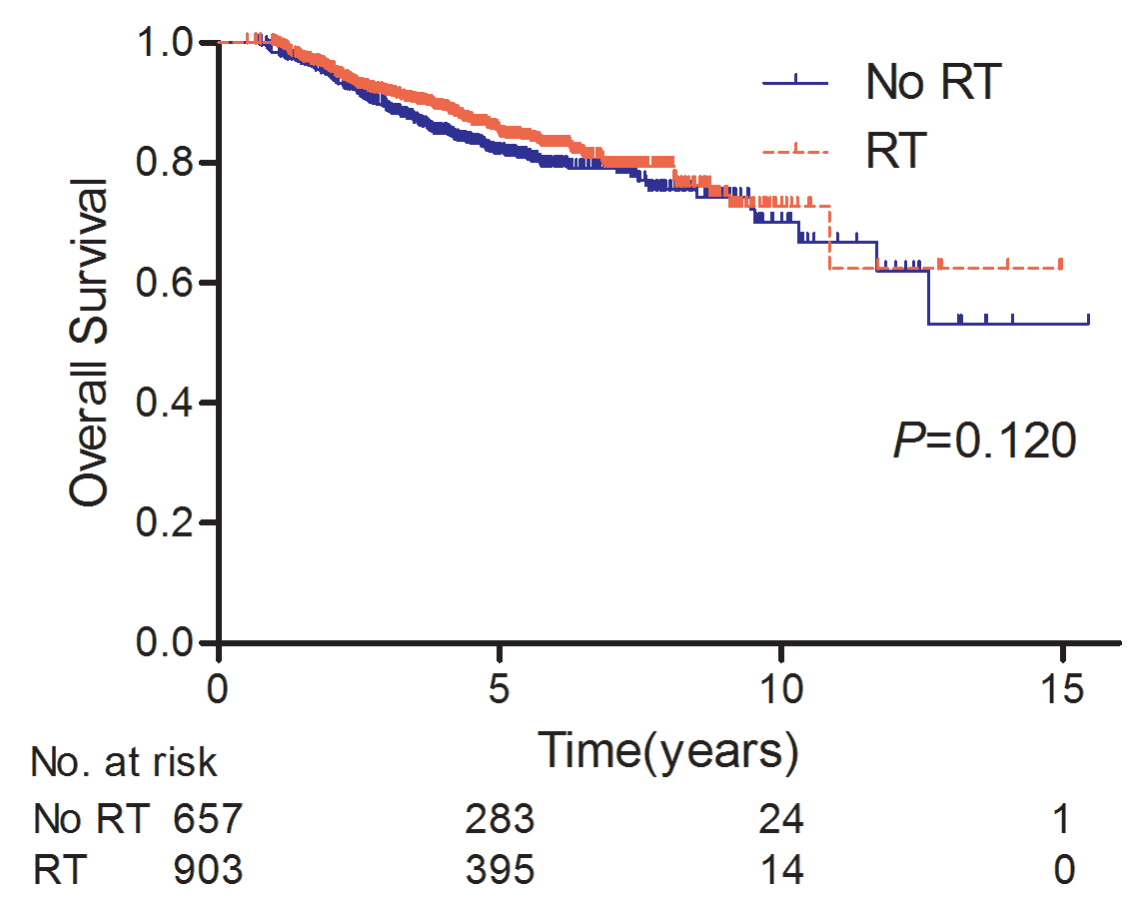
Rate of overall survival for the entire cohort of patients treated with PMRT (*n* = 903) and without PMRT (*n* = 657)

Overall, the median follow-up was 56.0 months (range, 6.14-185.4 months). At the cutoff date for the survival analysis (December 2013), a total of 139 (15.4%) and 124 (18.9%) patients died in the PMRT and no PMRT group, respectively. The 5-year OS rates in the two groups were not significantly different (84.6% for PMRT *vs* 81.7% for no PMRT, *P* = 0.120, Figure 1). PMRT also showed no association with a difference in OS by multivariate analysis (PMRT *vs* no PMRT: HR 0.820, 95% CI 0.630-1.068, Table 2). Factors found to be significant for worse OS by multivariate analysis included: age older than 60 years, white or black race, public insurance (compared with private insurance), higher histologic grade, fewer than 10 axillary nodes examined, clinical T4 tumor, clinical stage III disease, residual pathologic T2 tumor, and lack of hormone therapy (*P* < 0.05 for all comparisons, Table 2).

**Table 2:** Multivariate analysis of OS for the whole study population (*n* = 1560)

Factors	HR	95% CI	*P*
Age, years			
≤40	Reference		
41-60	1.209	0.857-1.706	0.281
>60	1.692	1.122-2.553	**0.012**
Race			
White	Reference		
Black	0.965	0.701-1.329	0.829
Asian or other	0.394	0.174-0.894	**0.026**
Insurance status			
Private insurance	Reference		
Public insurance	1.468	1.093-1.971	**0.011**
Not insured	1.155	0.645-2.068	0.627
Unknown	1.176	0.423-3.270	0.756
Histologic grade			
Well differentiated	Reference		
Moderately differentiated	9.749	1.331-71.425	**0.025**
Poorly or undifferentiated	7.760	1.066-56.489	**0.043**
Unknown	9.221	1.239-68.657	**0.030**
Examined regional nodes number			
0-10	Reference		
>10	0.770	0.598-0.991	**0.043**
Unkown	1.196	0.576-2.482	0.631
Clinical T-stage			
T1	Reference		
T2	0.692	0.419-1.141	0.149
T3	1.575	0.784-3.167	0.202
T4	2.808	1.395-5.649	**0.004**
Clinical AJCC stage			
II	Reference		
III	2.193	1.197-4.017	**0.011**
Pathologic T-stage (after NAC)			
T0/Tis	Reference		
T1	1.275	0.943-1.724	0.115
T2	1.599	1.160-2.205	**0.004**
Hormone therapy			
No	Reference		
Yes	0.647	0.441-0.951	**0.027**
Unknown	0.618	0.300-1.273	0.192
PMRT			
No	Reference		
Yes	0.820	0.630-1.068	0.141

Abbreviations: OS, overall survival; HR, hazard ratio; CI, confidence interval; AJCC, American Joint Committee on Cancer (fifth or sixth edition); NAC, neoadjuvant chemotherapy; PMRT, postmastectomy radiotherapy

**Figure 2:**
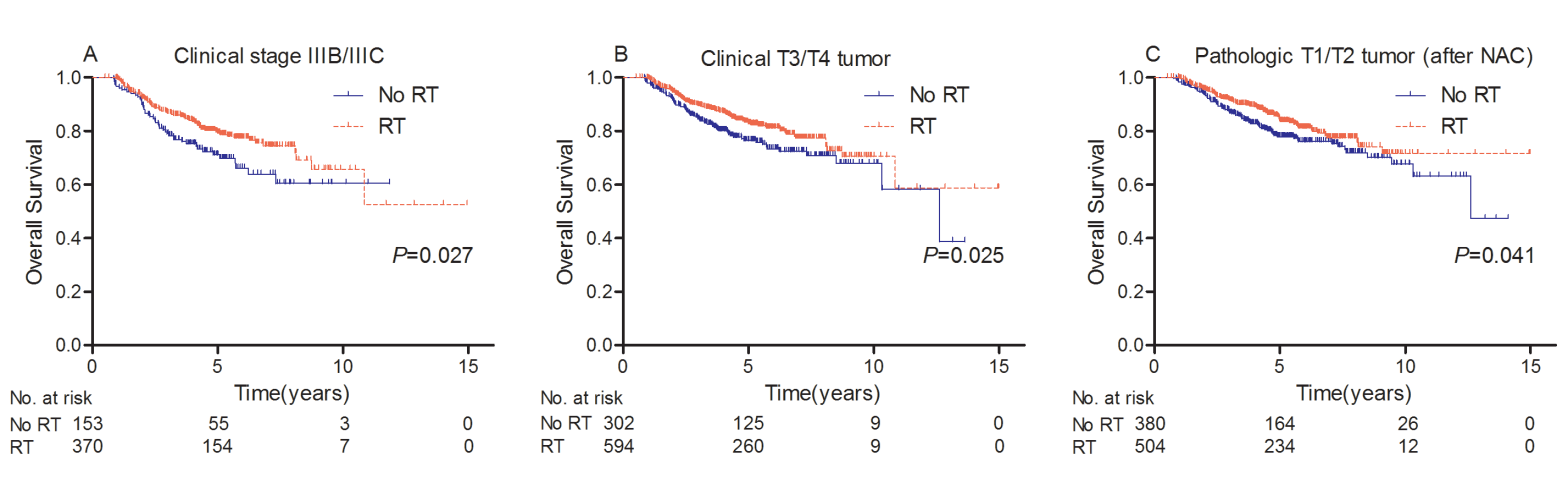
**Rate of overall survival for patients with A.** clinical IIIB/IIIC disease, **B.** clinical T3/T4 tumor, or **C.** pathologic T1/T2 tumor after NAC who were treated with PMRT and without PMRT.

However, subgroup analyses demonstrated PMRT significantly improved OS in patients with clinical stage IIIB/IIIC disease or T3/T4 tumor, or residual invasive breast tumor after NAC (*P* < 0.05 for all comparisons; Table 3, Figure 2A to 2C).

**Table 3:** Subgroup analyses for the effect of PMRT on the 5-year OS rate in the primary analysis

Factors	5-year OS Rate (%)		*P*
	No PMRT	PMRT	
Age			
≤40	90.2	86.7	0.136
41-60	82.8	85.4	0.151
>60	67.8	78.9	0.053
Clinical AJCC stage			
II	83.9	86.5	0.424
IIIA	86.8	89.5	0.247
IIIB/IIIC	71.2	79.3	**0.027**
Clinical T-stage			
T1/T2	86.0	87.8	0.329
T3/T4	76.6	82.8	**0.025**
Clinical N-stage			
N1	81.7	84.8	0.191
N2/N3	81.5	84.0	0.359
Pathologic T-stage (after NAC)			
T0/Tis	86.9	86.0	0.891
T1/T2	78.0	83.6	**0.041**
Hormone receptor^*^			
Negative	82.6	83.4	0.587
Positive	82.9	87.6	0.178

Abbreviations: PMRT, postmastectomy radiotherapy; OS, overall survival; AJCC, American Joint Committee on Cancer (fifth or sixth edition); NAC, neoadjuvant chemotherapy

* We created a joint hormone receptor status using estrogen receptor (ER) and progesterone receptor (PR) status. Those with either ER or PR positive status (ER or PR positive groups included those with borderline results) were grouped as hormone receptor positive, and those with ER and PR negative status were grouped as hormone receptor negative.

### Survival analyses for propensity score-matched populations

Since patients who received PMRT differed from patients who did not receive PMRT, we performed propensity score analysis as a sensitivity analysis to ensure that previous results were not due to lack of baseline covariate balance. Before the propensity score analysis, we used multiple imputation methods to account for the missing values of several variables. Sensitivity analysis showed that the effects of PMRT on OS were quite similar before and after imputation (data not shown).

Of the entire study population, we matched 523 pairs of patients by propensity scores. Table 4 shows the characteristics and standardized mean differences of all covariates between the two groups before and after matching. Univariate and multivariate analyses consistently showed no statistical effect of PMRT on OS (*P* = 0.167; PMRT *vs* no PMRT: HR 0.847, 95% CI 0.632-1.136, Supplementary Figure 1, and Supplementary Table 1). We also matched the patients who received PMRT to those who did not, by propensity scores among distinct subgroups of patients based on the above primary subgroup survival analyses. Subgroup analyses for propensity score-matched populations identified PMRT improved OS in patients with clinical stage IIIB/IIIC disease, T3/T4 tumor, or residual invasive breast cancer after NAC, which is the same subset of patients as those defined by the primary subgroup analyses (*P* < 0.05 for all comparisons; Table 5, Supplementary Figure 2A to 2C).

**Table 4:** Characteristics and standardised mean differences of covariates among patients with or without PMRT before and after propensity-score matching

Characteristics	Before matching (*n* = 1560)			After mathching (*n* = 1046)		
	No PMRT (%)	PMRT (%)	Std. mean difference	No PMRT (%)	PMRT (%)	Std. mean difference
Age, years						
≤40	21.8	22.5	0.017	22.0	22.7	0.018
41-60	58.7	60.3	0.032	58.5	60.4	0.039
>60	19.5	17.2	−0.060	19.5	16.9	−0.069
Race						
White	75.2	76.7	0.036	75.7	74.0	−0.041
Black	18.4	17.9	−0.012	18.4	20.5	0.055
Asian or other	6.4	5.3	−0.047	5.9	5.5	−0.017
Insurance status						
Not insured	5.1	5.6	0.054	5.7	5.3	0.017
Private insurance	66.4	68.9	0.020	66.9	67.7	−0.017
Public insurance	28.5	25.5	−0.069	27.4	27.0	−0.009
Chalson/Deyo score						
0	84.0	88.3	0.132	87.6	87.2	−0.012
1	6.7	9.2	0.086	7.5	8.6	0.040
2	1.5	0.8	−0.085	1.1	1.1	0.000
Unknown	7.8	1.8	−0.454	3.8	3.1	−0.058
Year of diagnosis						
1998-2003	13.7	6.2	−0.311	9.2	8.6	−0.024
2004-2009	86.3	93.8	0.311	90.8	91.4	0.024
Histological type						
Ductal	82.2	79.6	−0.064	81.2	81.4	0.005
Lobular	7.6	6.7	−0.039	7.3	7.3	0.000
Other	10.2	13.7	0.103	11.5	11.3	−0.006
No. of nodes examined						
1-10	49.8	42.9	−0.140	49.0	45.1	−0.077
>10	50.2	57.1	0.140	51.0	54.9	0.077
Clinical T-stage						
T1	12.0	6.1	−0.248	8.8	8.6	−0.008
T2	42.0	28.1	−0.309	39.0	39.2	0.004
T3	25.9	30.9	0.109	28.5	27.9	−0.012
T4	20.1	34.9	0.310	23.7	24.3	0.012
Clinical N-stage						
N1	80.7	72.1	−0.191	76.9	76.9	0.000
N2	13.7	17.8	0.108	16.0	14.5	−0.040
N3	5.6	10.1	0.148	7.1	8.6	0.051
Clinical AJCC stage						
II	49.5	25.6	−0.547	41.9	40.3	−0.035
III	50.5	74.4	0.547	58.1	59.7	0.035
Pathologic T-stage (after NAC)						
T0/Tis	42.2	44.2	0.041	43.2	40.9	−0.046
T1	33.6	34.9	0.026	33.7	34.0	0.008
T2	24.2	20.9	−0.080	23.1	25.1	0.047
Histologic grade						
Well	2.7	2.7	−0.005	2.5	2.9	0.024
Moderatedly	25.3	22.1	−0.075	26.0	25.6	−0.009
Poorly or undifferentiated	72.0	75.2	0.074	71.5	71.5	0.000
ER^*^						
Negative	50.2	55.7	0.110	53.7	51.8	−0.038
Positive	31.7	36.7	0.104	34.6	37.1	0.052
Unknown	18.1	7.6	−0.394	11.7	11.1	−0.022
PR^*^						
Negative	57.7	62.3	0.096	61.2	59.5	−0.035
Positive	24.2	29.9	0.124	27.2	29.2	0.046
Unknown	18.1	7.8	−0.387	11.6	11.3	−0.014
Chemotherapy type						
Single-agent	3.0	0.5	−0.391	0.6	0.8	0.029
Multi-agent	97.0	99.5	0.391	99.4	99.2	−0.029

Abbreviations: PMRT, postmastectomy radiotherapy; NS, not significant (*P* >0.05);

AJCC, American Joint Committee on Cancer (fifth or sixth edition); NAC, neoadjuvant chemotherapy; ER, estrogen receptor; PR, progesterone receptor

* ER or PR groups include those with borderline results.

**Table 5:** Subgroup analyses for the effect of PMRT on the 5-year OS rate in the sensitivity analysis (for the propensity score-matched patients)

Factors	5-year OS Rate (%)		*P*
	No PMRT	PMRT	
Age			
≤40	92.3	87.4	0.296
41-60	82.8	86.8	0.076
>60	67.8	77.8	0.138
Clinical AJCC stage			
II	80.7	86.0	0.236
IIIA	53.1	51.9	0.687
IIIB/IIIC	71.6	80.4	**0.046**
Clinical T-stage			
T1/T2	85.1	85.5	0.420
T3/T4	76.9	81.7	**0.049**
Clinical N-stage			
N1	83.4	84.9	0.247
N2/N3	81.2	80.3	0.946
Pathologic T-stage (after NAC)			
T0/Tis	91.2	86.0	0.645
T1/T2	78.0	84.6	**0.032**
Hormone receptor^*^			
Negative	83.7	80.4	0.580
Positive	81.5	85.6	0.352

Abbreviations: PMRT, postmastectomy radiotherapy; OS, overall survival; AJCC, American Joint Committee on Cancer (fifth or sixth edition); NAC, neoadjuvant chemotherapy

* We created a joint hormone receptor status using estrogen receptor (ER) and progesterone receptor (PR) status. Those with either ER or PR positive status (ER or PR positive groups included those with borderline results) were grouped as hormone receptor positive, and those with ER and PR negative status were grouped as hormone receptor negative.

## DISCUSSION

NAC is being used more frequently for clinical stage II or III breast cancer patients, raising issues regarding to the subsequent locoregional treatment, such as radiotherapy and sentinel node biopsy. However, the role of PMRT in clinically node-positive, stage II-III patients with negative pathological nodes (ypN0) after NAC, remains unclear. The current study included a large, registry-based, national patient cohort, with the aim to address this question. Our findings suggest that PMRT improves patient OS, but its benefit appears to be limited to selected patients with clinical stage IIIB/IIIC disease, clinical T3/T4 tumor, or residual invasive breast tumor after NAC.

One of the main effects of NAC is its potential to downstage the pathological extent of disease. Previous studies showed 20% to 40% of breast cancer patients with clinically positive nodes at diagnosis can achieve a complete pathological nodal response after NAC [20, 21]. This pathological downstaging presents a unique challenge to treatment decision-making. Further complicating the issue is the lack of definitive data in the literature. Studies regarding the effectiveness of PMRT among women with clinically node-positive, stage II-III disease who downstaged to ypN0 following NAC were all based on retrospective analysis of small patient cohorts, and had conflicting results. A study from MD Anderson Cancer Center found that PMRT reduced LRR for patients with clinical stage III or IV disease and subsequently achieved a pCR to NAC, but no difference in LRR rates was observed in patients with clinical stage I-II disease with a pCR [4]. Their updated single-institutional experience, including 106 patients with a pCR in the breast and regional lymph nodes to NAC, indicated similar results (selected patients with clinical stage III disease with a pCR after NAC can benefit from PMRT) [11]. However, a French study showed that PMRT was not associated with improved local recurrence-free survival, disease-free survival or OS in women with ypN0 after NAC [9]. Similarly, a multicenter retrospective study (*n* = 151) also reported that PMRT had no effectiveness in clinical stage II-III Korean breast cancer patients with ypN0 following NAC[10]. Since radiotherapy for breast cancer has toxicities including cardiac complications, pneumonitis, and lymphedema [22], and PMRT may lead to additional plastic surgeries for completion of breast reconstruction, the authors recommended the omission of PMRT in clinical stage II-III patients with ypN0 after NAC. Our study, based on a large patient cohort, on the other hand, is in support with the observations made by the MD Anderson studies. Unlike the French and Korean study, which had inherent shortcomings such as limited sample size, and multiple unbalanced baseline characteristics between groups, the current study represent the largest and utilized the most contemporary analysis to address this issue. The large sample size and more robust sensitivity analyses using imputation and propensity score matching enabled us to better quantify the survival benefit of PMRT for multiple subgroups of patients with minimal potential biases.

Clinically node-positive, stage II-III breast cancer patients who had complete nodal response to NAC after mastectomy represent a heterogeneous collection of patients with a wide range of demographic, clinicopathologic and treatment response related characteristics. Therefore, an important question to ask is which specific subset of these patient would most benefit from additional PMRT. Despite clinical disease stage before NAC, are there any other factors (such as ER/PR status, or pathologic tumor stage after NAC) will influence the patient selection for PMRT among these ypN0 women? A combined analysis of two National Surgical Adjuvant Breast and Bowel Project (NSABP) trials (B-18 and B-27) concluded that in addition to initial tumor characteristic before NAC, pathologic response in the breast and the axillary lymph nodes had a major impact on the rates and patterns of LRR; so the pathologic tumor stage and nodal status after NAC might be useful factors for predicting the optimal use of postmastectomy radiation in patients treated with preoperative chemotherapy [23]. Other studies reported that lymphovascular invasion (LVI) was another possible effect modifier that can aid in selecting patients who have a ypN0 following NAC but would benefit from PMRT [9, 24]. Based on our findings, it appears that patients with more locally advanced disease at presentation (clinical IIIB/IIIC disease or T3/T4 tumor), or with residual invasive breast tumor after NAC (who did not achieve a pCR in the breast) may benefit PMRT as part of their adjuvant therapy even if they downstaged to ypN0 following NAC. However, despite our best attempt to reduce the potential bias and confounding effects from NCDB and the retrospective nature of our study, definitive evidence from randomized controlled trials like NSABP B-51 are required to confirm our results, or identify further subsets of patients who may benefit from PMRT.

Despite several strengths of this study including its multicenter large sample size, refined subgroup analyses, sensitivity analysis using multiple imputation and propensity score matching, several limitations should be acknowledged. First, the NCDB has no recurrence data, so we can not affirm a lack of benefit from PMRT for some subgroups of women simply based on OS alone. This is especially the case for patients with earlier clinical stage disease, where disease control and free from recurrence would more likely be the primary endpoint of interest. Second, the NCDB suffers from lack of HER2 status and detailed histological evaluation including the information of LVI, Ki67, and the incompleteness of ER/PR status in a small portion of patients. All these factors are known to have prognostic value and can predict treatment response. Third, because of the limited number of patients (reduced statistical power) in several subgroup analyses, we cannot conclude lack of survival benefit from PMRT, particularly for patients who were diagnosed before 41 years or after 60 years. Finally, although our study has a median follow-up time of 56.0 months, longer follow-up might help explore other subsets of women who can benefit from PMRT, especially for low-risk patients.

## CONCLUSIONS

In conclusion, we provided important evidence that among clinically node-positive, stage II-III breast cancer patients with ypN0 following NAC, PMRT can improve overall survival in patients with clinical T3/T4 tumor or stage IIIB/IIIC disease, and in patients with residual invasive breast tumor after NAC. Our study may help oncologists to recommend PMRT for selected patients who downstaged to ypN0 following NAC. Results from further prospective studies such as the ongoing NSABP B-51 trial are needed, in order to confirm our findings and define other specific subgroups of women with pathological negative nodes following NAC who would benefit from PMRT, particularly in the relatively low-risk patients.

## SUPPLEMENTARY MATERIAL FIGURES AND TABLE


