# Supplementary material for: The role of postmastectomy radiotherapy in clinically node-positive, stage II-III breast cancer patients with pathological negative nodes after neoadjuvant chemotherapy: an analysis from the NCDB
Source: Oncotarget. 2015 Dec 18;7(17):24848–59. doi: 10.18632/oncotarget.6664 (PMC5029747; doi:10.18632/oncotarget.6664)
Supplement: Supplementary file 1 [file oncotarget-07-24848-s001.pdf]

The role of postmastectomy radiotherapy in clinically node-positive, stage II-III breast cancer patients with pathological negative nodes after neoadjuvant chemotherapy: an analysis from the NCDB

Supplementary Material

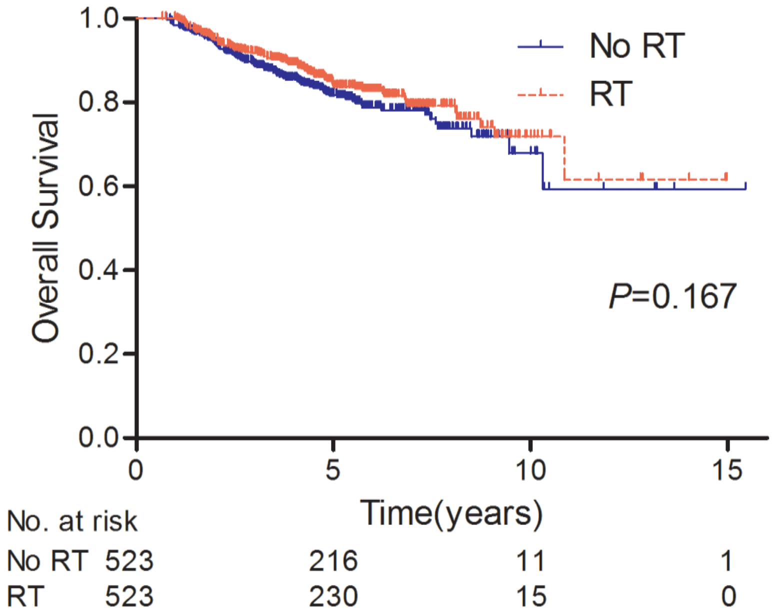

Supplementary Figure 1 Rate of overall survival for the propensity score-matched patients treated with PMRT (n=523) and without PMRT (n=523).

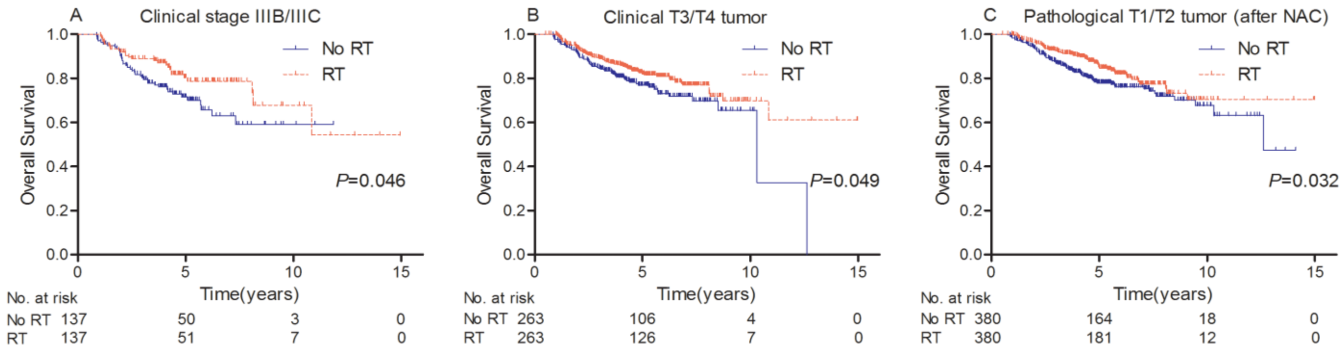

Supplementary Figure 2 Rate of overall survival for the propensity score-matched patients with A) clinical IIIB/IIIC disease, B) clinical T3/T4 tumor, or C) pathologic T1/T2 tumor after NAC who were treated with PMRT and without PMRT.

Supplementary Table 1 Multivariate analysis of PMRT's effect on OS for the propensity score-matched patients (n=1046)

Factors not included in the propensity score	HR	95% CI	*P*
Hormone therapy			
No	Reference		
Yes	0.594	0.423-0.833	**0.003**
PMRT			
No	Reference		
Yes	0.847	0.632-1.136	0.268

Abbreviations: PMRT, postmastectomy radiotherapy; OS, overall survival; HR, hazard ratio; CI, confidence interval

Abbreviations: OS, overall survival; HR, hazard ratio; CI, confidence interval; AJCC, American Joint Committee on Cancer (fifth or sixth edition); NAC, neoadjuvant chemotherapy; PMRT, postmastectomy radiotherapy
